# Malnutrition in Older Hip Fracture Patients: Prevalence, Pathophysiology, Clinical Outcomes, and Treatment—A Systematic Review

**DOI:** 10.3390/jcm14165662

**Published:** 2025-08-11

**Authors:** Geert Meermans, Jeroen C. van Egmond

**Affiliations:** Department of Orthopaedics, Bravis Hospital, Boerhaavelaan 25, 4708 AE Roosendaal, The Netherlands; j.vanegmond@bravis.nl

**Keywords:** malnutrition, hip fractures, older patients, geriatric, sarcopenia, nutritional assessment, oral nutritional supplementation

## Abstract

**Background**: Malnutrition is highly prevalent among older patients with hip fractures and significantly impacts recovery and survival. This narrative review synthesizes current evidence on the prevalence, pathophysiology, and clinical consequences of malnutrition in hip fracture patients, along with diagnostic tools and nutritional interventions. **Methods**: A literature search of studies from 2000 to 2025 identified consistent associations between malnutrition—defined using tools such as the Mini Nutritional Assessment (MNA), Geriatric Nutritional Risk Index (GNRI), and serum albumin levels—and increased risks of postoperative complications, prolonged hospital stays, functional decline, and mortality. Pathophysiological mechanisms include sarcopenia, systemic inflammation, and impaired bone metabolism. **Results**: Notably, malnutrition is associated with fracture type, with low lean body mass and poor nutritional status correlating with intracapsular femoral neck fractures. **Conclusions**: Interventional studies demonstrate that oral nutritional supplementation (ONS) reduces complications and improves biochemical parameters but shows mixed effects on long-term mortality and function. The findings support routine nutritional screening and early intervention in older hip fracture patients to improve outcomes and reduce the healthcare burden.

## 1. Introduction

Hip fracture represents a major public health challenge in geriatric populations due to its high incidence, significant morbidity, and mortality [1,2]. In women, the estimated lifetime risk is approximately 14%, compared with 6% in men [3]. Due to an aging population, the burden is expected to increase further in the upcoming decades. A Dutch study found the overall incidence rate of femoral neck and trochanteric fractures increased by 22% over a 20-year period [4]. Globally, age- and sex-standardized hip fracture incidence rates have declined in recent years in most countries and regions [5]. However, in the same study, the authors found that as the global population ages, the burden of hip fractures will increase over time, with the absolute number of hip fractures projected to double by 2050 from nearly 1 million globally in 2018 to nearly 2 million in 2050.

In older hip fracture patients, the reported prevalence of malnutrition is highly variable, depending on the geographical location and the assessment tools used [6]. The coexistence of malnutrition and hip fractures has profound clinical implications. Malnutrition significantly increased the risk of adverse outcomes in patients with a fracture including postoperative delirium, mortality, mobility, pressure ulcers, length of stay (LOS), and the need for more intensive living support [6,7,8,9].

The pathophysiological interplay between malnutrition and hip fracture outcomes is multifactorial. Under-nourished patients often suffer muscle wasting or sarcopenia coupled with low bone mineral density (BMD), predisposing them to falls and fractures. Post-fracture, systemic inflammation and catabolism further increase nutritional demands while pain and physical limitations reduce intake [10,11]. Biomarkers such as low BMI, albumin, and elevated oxidants have been consistently linked to poorer functional recovery, greater complications, and higher mortality [12,13].

Encouragingly, interventional trials indicate that targeted nutritional support through supplementation or dietary counseling improves outcomes [12,13]. Older patients receiving nutritional interventions show enhanced functional recovery and reduced complications, supporting current ESPEN guidelines recommending routine supplementation for older hospitalized hip fracture patients [14].

Despite compelling evidence, gaps remain concerning the optimal screening protocols, timing, and nature of nutritional interventions. This narrative review was conducted to synthesize current evidence on the prevalence of malnutrition in hip fracture patients, quantify its impact on the type of fracture and key outcomes (mortality, functional recovery, and LOS), evaluate the different assessment methods, and consider the efficacy of early nutritional strategies in improving postoperative trajectories.

## 2. Materials and Methods

### 2.1. Search Strategy

This systematic review was performed based on the Preferred Reporting Items for Systematic Reviews and Meta-Analyses (PRISMA) checklist [15]. A comprehensive literature search was performed using PubMed, Scopus, and Web of Science databases. The search spanned from January 2000 to April 2025. The following search terms were used in combination with Boolean operators: (“malnutrition” OR “nutritional status” OR “undernutrition” OR “nutritional assessment”) AND (“hip fracture” OR “femoral neck fracture” OR “neck of femur fracture” OR “intertrochanteric fracture” OR ”pertrochanteric fracture” OR “proximal fem* fracture”) AND (“prevalence” OR “mortality” OR “rehabilitation” OR “functional outcome” OR “complication*” OR “nutritional intervention” OR “review” OR “cohort” OR “randomized”). Searches were limited to articles published in English and involving human subjects aged ≥60 years. Additional sources were identified by manually searching references of relevant systematic reviews and meta-analyses.

### 2.2. Inclusion and Exclusion Criteria

Eligible studies included original research articles—prospective and retrospective cohort studies, case-control studies, randomized controlled trials, and systematic reviews—that examined the role of malnutrition in the context of hip fractures. Studies were included if they (1) assessed nutritional status using validated measures (e.g., Mini Nutritional Assessment (MNA), serum albumin, body mass index (BMI), Geriatric Nutritional Risk Index (GNRI)), and (2) reported clinical outcomes such as mortality, complication rates, hospital LOS, or functional recovery. Exclusion criteria were as follows: non-English publications, conference abstracts without full data, studies involving patients under 60 years of age, studies focused exclusively on pediatric or trauma populations unrelated to hip fractures, and papers that assessed nutritional status without linking it to outcomes.

### 2.3. Data Synthesis

Findings were summarized descriptively. Due to heterogeneity in study design, populations, and outcome measures, meta-analyses were not performed. Instead, results were synthesized narratively with a focus on the prevalence of malnutrition, its association with clinical outcomes, and the reported impact of nutritional interventions in the hip fracture setting.

## 3. Results

**Figure 1:**
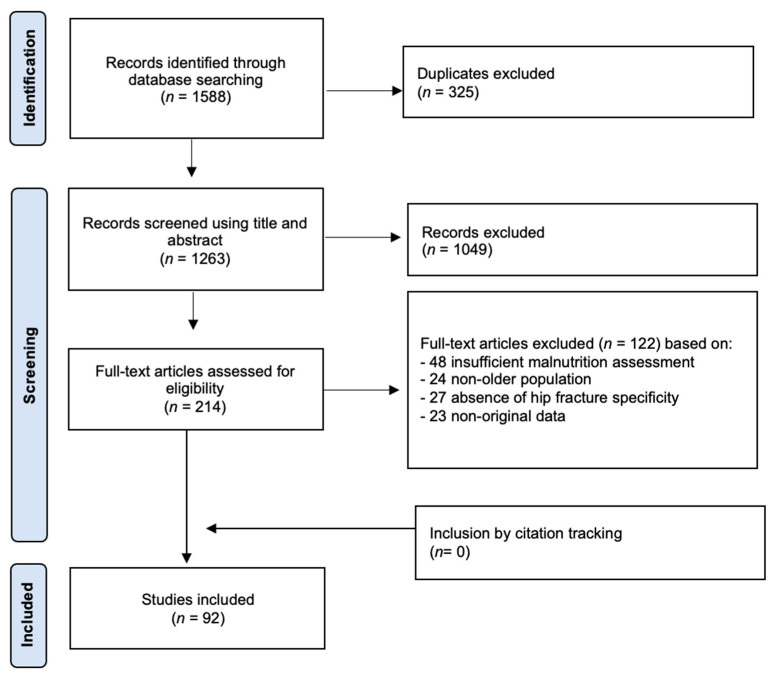
Flow diagram of the systematic literature search on malnutrition in older patients with hip fractures.

A comprehensive search was conducted across the PubMed, Scopus, and Web of Science databases to identify studies investigating the relationship between malnutrition and hip fractures in older populations. After the removal of duplicates, a total of 1263 records were screened based on their titles and abstracts. Following preliminary screening, 214 full-text articles were assessed for eligibility. Ultimately, 92 studies were included in the qualitative synthesis based on predefined inclusion and exclusion criteria (Figure 1). Full-text articles were excluded for having insufficiently detailed malnutrition assessments (*n* = 48), having populations other than older patients (*n* = 24), lacking hip fracture specificity (*n* = 27), and containing non-original data (e.g., editorials and reviews) (*n* = 23).

A total of 19 studies reported prevalence estimates using validated nutritional screening tools, including the Mini Nutritional Assessment (MNA), Subjective Global Assessment (SGA), and the GLIM criteria. Thirteen studies explored the biological and physiological mechanisms linking malnutrition with impaired healing and systemic deterioration post-hip fracture. Eleven studies examined whether malnutrition status influenced fracture type. Twenty-eight studies assessed the impact of malnutrition on healing time, complications, and long-term recovery. Fifteen studies evaluated nutritional assessment tools in hip fracture cohorts. Twenty-two interventional studies explored the efficacy of in-hospital nutritional support.

**Summary of Findings**

### 3.1. Prevalence

The World Health Organization defines malnutrition as “deficiencies, excesses or imbalances in a person’s intake of energy and/or nutrients” [16]. Nutritional status has been determined by serum markers as well as anthropometric measurements. Abnormalities in any of these values can be considered as malnutrition and may impair outcomes. Malnutrition is common among older adults admitted with hip fractures. Several robust hospital-based studies assessing nutritional status within the initial 24–48 h of hospital admission consistently indicate a wide range of the prevalence of malnutrition, generally between 8% and 52%, although most findings cluster within the 15–40% range [6,12,17,18,19,20,21,22,23,24,25,26,27,28,29,30,31,32,33].

A narrative review of 44 studies [12] involving 26,281 patients (with a mean age of 83.6 ± 7.2 years; 73.5% of whom were women) found that 18.7% were malnourished according to the MNA long or short form (a range of 0–63.6%). This prevalence was even higher (45.7%; range: 12–45.9%) when malnutrition was defined using broader criteria such as low BMI, weight loss, or hypoalbuminemia. A more recent systematic review, using more stringent inclusion criteria, found similar results [6]. They included nine studies with 1665 patients with a mean age that ranged from 79.9 to 86.1 years and a majority of female patients (with a range from 70% to 81.8%). Among the established nutrition assessment tools, only the Subjective Global Assessment (SGA) and the MNA were used in the eligible studies. The study that used the SGA reported a malnutrition prevalence of 39.4%, whereas the studies that used the MNA reported a malnutrition prevalence ranging from 4% to 28%.

This variability underscores the influence of diagnostic method on reported prevalence and highlights that substantial numbers of malnourished patients may remain undetected without a comprehensive assessment. Nonetheless, both the SGA and the MNA were found to have similar prognostic abilities with a low risk of bias in this aforementioned systematic review. Timing of assessment also critically influences the accuracy of prevalence data. Nutritional evaluations conducted immediately upon or within 48 h of hospital admission most accurately reflect baseline nutritional status, avoiding the confounding influence of acute surgical stress, postoperative metabolic responses, and inflammation-induced hypoalbuminemia, which commonly occur in hip fracture contexts [12]. Studies that delay assessment beyond the initial 48 h frequently report misleading prevalence data, which are either inflated or deflated due to acute physiological fluctuations [34].

Despite the wide ranges, the prevalence of malnutrition in older patients with hip fractures is higher than in community-dwelling older adults [35,36,37]. The observed variability in the prevalence of nutritional status in hip fracture patients arises from differences in study design, population demographics (e.g., age, sex, care setting, and geographics), timing of assessment (prehospital vs. perioperative), and the lack of a universal consensus as to the best measure to diagnose protein-energy malnutrition. This lack of universality limits the comparison of the various studies, making it difficult to carry out a consistent malnutrition diagnosis. This could delay the clinical decision to prescribe nutritional treatment in some of these patients.

### 3.2. Pathophysiology

A central mechanism is sarcopenia, an age-related loss of muscle mass and strength. In hip fracture patients, sarcopenia is highly prevalent and is strongly associated with indicators of malnutrition such as low BMI, hypoalbuminemia, and hypoproteinemia [38,39,40,41]. These deficits are often further exacerbated by inadequate dietary intake in the pre-hospital period. For instance, hospitalized patients on average consumed only ~930 kcal/day and ~0.9 g protein/kg/day, insufficient for maintaining their muscle mass, and therefore, this nutritional deficit correlated with reduced muscle mass measured via bioelectrical impedance [42].

Protein-energy malnutrition is also nearly universal in acute hip fracture cases. A cohort of 509 patients revealed that 81% had biochemical evidence of protein malnutrition (albumin < 3.5 g/dL or total protein < 6.5 g/dL), and over 90% exhibited vitamin D deficiency, factors that compromise muscle and bone integrity [43]. The inflammatory response triggered by fracture and surgery amplifies metabolic demands while simultaneously increasing catabolism. Chronic inflammation is prevalent in sarcopenic hip fracture patients and drives muscle protein degradation via ubiquitin–proteasome and autophagy pathways [44]. Oxidative stress further exacerbates muscle damage and impairs satellite-cell-mediated repair.

Simultaneously, malnutrition contributes to bone demineralization. Protein-energy deficits reduce serum IGF-1, a critical anabolic factor for bone and muscle tissue, thereby accelerating osteoporosis and elevating fracture risk [45]. Hormonal imbalances increase these effects: ageing increases cortisol-to-DHEA ratios, suppresses anabolic hormones like IGF-1 and testosterone, and downregulates mTOR signaling, cumulatively leading to frailty and a reduced physiological reserve.

Moreover, fracture patients enter a state of hypercatabolism, driven by systemic inflammatory response and tissue repair demands, while their intake remains suboptimal due to the pain, immobility, and anorexia that stem from ageing [12,46]. These factors sustain low albumin levels, a reduced antioxidant capacity, and heightened free-radical production, all linked to longer hospital stays and elevated complication rates.

Cognitive impairment, including dementia and delirium, significantly compounds the nutritional risks faced by older hip fracture patients. Dementia, characterized by progressive cognitive decline, is strongly associated with baseline malnutrition due to factors such as reduced appetite, impaired swallowing mechanisms, and a diminished ability to independently access and consume nutritious food [22,47,48]. Cognitive impairment also leads to difficulties in communicating nutritional needs, adhering to dietary interventions, and recognizing symptoms of nutritional deficiencies, thereby delaying or complicating clinical management and recovery [49].

This confluence of factors—reduced intake, increased energy and protein expenditure, hormonal dysregulation, inflammation, and oxidative stress—creates a detrimental cycle: malnutrition leads to worsened sarcopenia and bone fragility, elevating fracture risk and compromising post-fracture recovery. Breaking this cycle requires early, targeted nutritional interventions aimed at protein-calorie replenishment, anti-inflammatory strategies, vitamin D and micronutrient repletion, and the incorporation of resistance exercise to restore muscle and bone function.

### 3.3. Influence on Fracture Type

The type of hip fracture sustained by older patients has significant clinical implications and appears to be closely related to nutritional status. The fracture risk is jointly determined by the risk of fall and the load–strength ratio [50]. A two-level subject-specific biomechanical finite element model demonstrated that underweight individuals, with low BMI and diminished soft tissue, exhibited an increased predicted fracture risk from sideways falls. Subjects with a higher BMI usually have thicker soft tissues over their hip, which is able to greatly attenuate the impact energy and thus reduce the impact force. In addition, BMD is associated with BMI, which means subjects with a higher BMI generally have stronger bones, and they are thus able to withstand higher impact forces without fracture [51]. A fall-simulation study combined DEXA and trochanteric soft-tissue thickness measures in women. A lower BMI and reduced soft tissues over the hip correlated with higher femoral neck stresses during sideways falls, explaining why leaner individuals are more prone to femoral neck fractures [52]. This confirms earlier epidemiological observations that a low body weight increases hip fracture risk through higher focal stress on the femoral neck [53,54].

A case-control study comparing older patients with femoral neck fractures to non-fractured controls demonstrated that those with neck fractures had significantly lower levels of serum albumin and prealbumin. Bioelectrical impedance analysis revealed markedly reduced appendicular lean mass, whereas fat mass differences were not significant. They concluded that muscle loss, not adiposity, was a critical risk factor for femoral neck fractures [55]. In a prospective cohort study on community-dwelling older women, of whom 279 had a femoral neck fracture and 222 had an intertrochanteric fracture of the proximal femur, the authors found that femoral neck fractures were largely predicted by BMD and poor functional ability, while aging and poor health status predisposed them to intertrochanteric fractures [56].

A literature review noted that patients with femoral neck fractures often present with a lower BMI, while intertrochanteric fracture patients tend to exhibit a higher BMI. This pattern supports the hypothesis that body composition differences—particularly low muscle mass—differentially influence fracture mechanics in femoral neck versus trochanteric regions [57]. Furthermore, in patients with a BMI > 30 that had an intertrochanteric fracture, there were more unstable intertrochanteric fracture patterns [58]. However, comprehensive comparative cohort data are sparse. A study using the Global Leadership Initiative on Malnutrition (GLIM) and Nutrition Risk Screening 2002 (NRS-2002) reported no significant difference in malnutrition prevalence between neck and trochanteric fracture types [59]. This may reflect overlapping nutritional risk across fracture types rather than negating subtype-specific influences.

In a more recent systematic review, patients with a pertrochanteric fracture were found to be older, with a low cortical thickness in the femoral isthmus and low BMD in the intertrochanteric region, and weighed less. Patients with femoral neck fractures tended to be younger and taller, with lower BMD levels in the femoral neck region [60]. Mechanistically, low lean body mass and protein malnutrition weaken the peri-articular musculature and reduce shock absorption during low-energy falls, increasing direct load transmission to the femoral neck cortex and thus susceptibility to intracapsular fractures. Conversely, higher fat and muscle mass in trochanteric regions may distribute mechanical forces differently, often resulting in fractures through the intertrochanteric cancellous bone.

### 3.4. Impact on Healing and Recovery

The impact of malnutrition on healing and recovery in older patients with hip fractures is profound, significantly affecting both immediate postoperative outcomes and long-term rehabilitation. Nutritional status, encompassing both protein-energy malnutrition and micronutrient deficiencies, directly influences the physiological processes necessary for effective wound healing, functional recovery, and overall survival post-hip fracture. One large database study analyzing 1667 hip fracture patients treated with total hip arthroplasty revealed that preoperative hypoalbuminemia (<3.5 g/dL) was associated with an 80 % increased risk of any postoperative complication (OR 1.80), a doubled risk of major complications (OR 2.13), and nearly doubled re-operation rates (OR 1.97) compared with well-nourished patients [61]. This emphasizes the broad adverse impact of poor nutritional status on surgical outcomes.

#### 3.4.1. Mortality

Malnutrition is a consistent and independent predictor of increased mortality among older patients who suffer a hip fracture during hospitalization, in the early postoperative period, and up to one year after the fracture. Patients identified as malnourished using tools such as the Mini Nutritional Assessment (MNA), the Geriatric Nutritional Risk Index (GNRI), and low serum albumin levels are shown to be at particularly elevated risk. The evidence spans a wide range of geographic and clinical settings, further supporting the robustness of this association [62,63,64].

Several studies found that malnourished patients had a two- to threefold increased risk of 1-year mortality compared to those with normal nutritional status [18,24,27,33,64,65,66,67,68,69,70,71,72,73,74,75,76]. This elevated risk persists even when adjusted for confounders such as age, gender, comorbidities, and fracture severity. Malnutrition was also associated with increased short-term mortality during acute hospitalization and within the first 30 days postoperatively. In some cohorts, hypoalbuminemia alone was found to be an equally strong predictor of mortality as age or comorbidity burden [7,8,12]. Furthermore, combined nutritional deficits—such as low BMI and low albumin—had a cumulative impact, with the highest mortality seen in those exhibiting both features [69,77].

Notably, cognitive impairment, common in this patient population, has been shown to exacerbate the effects of malnutrition on survival. Patients with dementia or delirium who were also malnourished demonstrated particularly poor outcomes. Similarly, frailty, which often coexists with malnutrition, further increased vulnerability to fatal complications, including pneumonia, sepsis, and thromboembolic events [70].

Importantly, the type of nutritional assessment used influenced mortality prediction. MNA and GNRI were found to be more sensitive to subtle nutritional risk and better predictors of long-term outcomes than single biochemical markers. Studies that stratified patients into well-nourished, at-risk, and malnourished groups revealed a stepwise increase in mortality across these categories [78,79].

#### 3.4.2. Length of Hospital Stay

Malnutrition has a well-documented association with a prolonged hospital length of stay (LOS) in older patients hospitalized for hip fracture. This relationship is multifactorial and arises from a combination of delayed surgical recovery, increased complication rates, impaired wound healing, and the need for more intensive perioperative support. Malnourished patients often present with poorer pre-fracture functional status and greater dependency, which further extends their recovery time. These patients are also more likely to experience medical complications such as infections, electrolyte imbalances, and cardiovascular instability, all of which prolong hospitalization [7,72].

Numerous studies have quantified the impact of malnutrition on LOS, with findings showing an increase of between 3 and 7 days on average in malnourished patients compared to their well-nourished counterparts [12,66,67,80,81]. Some studies identified malnutrition as the strongest independent predictor of hospital stays exceeding 10 days, even after adjusting for fracture type, surgical delay, and age. These effects appear to be most pronounced in patients with albumin levels below 3.5 g/dL or MNA scores below 17, suggesting that moderate to severe malnutrition has a particularly detrimental impact [80,82,83].

In addition to baseline nutritional status, inadequate postoperative intake is also a significant contributor to an extended LOS. Older hip fracture patients frequently experience reduced appetite, postoperative nausea, or difficulty feeding themselves—factors that exacerbate nutritional deficits acquired prior to hospitalization. Patients with the lowest caloric and protein intake in the first 72 h postoperatively have been found to remain hospitalized significantly longer than those meeting basic nutritional targets [84,85].

Malnourished patients were also more likely to require prolonged stays in step-down or rehabilitation units after discharge, adding to the total burden of care. Some studies estimated that malnutrition-related delays accounted for up to 20% of excess inpatient days in orthopedic wards treating older hip fracture patients [48]. The consistent association between poor nutritional status and extended LOS across different care settings and healthcare systems underscores the need for universal screening and nutritional intervention strategies.

#### 3.4.3. Functional Recovery

Malnutrition has a profound impact on functional recovery following hip fracture in older adults. Adequate nutrition is essential for tissue repair, muscle regeneration, and bone remodeling—all critical processes during the postoperative and rehabilitation periods. Malnourished individuals often present with pre-existing sarcopenia and frailty, which limit their capacity for physical recovery even before the fracture. These baseline deficits are exacerbated postoperatively, leading to slower mobilization, delayed weight-bearing, and reduced independence [33,65,66,76].

Across multiple studies, malnutrition was associated with significantly lower scores on functional assessments such as the Barthel Index, Functional Independence Measure (FIM), and walking tests [7,8,26,66,75,86]. Patients with low MNA or GNRI scores were less likely to regain pre-fracture mobility and more likely to remain dependent in activities of daily living (ADL). These patients also required longer rehabilitation periods and were more likely to be discharged to institutional care rather than to their homes. In some studies, the rate of recovery to independent ambulation was halved in malnourished patients compared to those with adequate nutrition [87,88,89].

Some studies also reported on the psychological impacts of malnutrition, including increased rates of depression and cognitive impairment, which themselves are barriers to effective rehabilitation. These secondary effects of malnutrition may reduce motivation, adherence to therapy, and patient engagement, thereby compounding physical limitations [73].

### 3.5. Nutritional Assessment Tools in Hip Fracture Care

Effective nutritional assessment is critical in hip fracture management to identify at-risk patients and guide timely interventions. Several validated screening tools—ranging from questionnaire-based instruments to index calculators incorporating biochemical markers—are routinely used. Among these, the Mini Nutritional Assessment (MNA), Geriatric Nutritional Risk Index (GNRI), Nutritional Risk Screening 2002 (NRS-2002), Prognostic Nutritional Index (PNI), and Controlling Nutritional Status (CONUT) scores have demonstrated relevance in hip fracture populations [90] (Table 1).

The MNA (including the full and short-form (MNA-SF) versions) is widely used in geriatrics and orthopedics and the most commonly used screening tool in hip fracture patients [8,12,26,27,71,91]. A prospective study of 86 patients aged >65 years showed that MNA administered within 72 h of hip fracture strongly predicted 6-month gait recovery and mortality, outperforming NRS-2002 and ASA scores [79]. Similarly, Inoue et al. [89] analyzed 205 hip fracture patients and looked at the association between each nutritional status assessed by four standard nutritional screening tools at admission and functional outcomes. The MNA-SF was found to be an optimal nutritional screening tool associated with functional outcomes during the postoperative acute phase of older hip fracture patients [89]. In contrast, no significant relationships were found for more general tools like MUST and NRS-2002. Furthermore, a population-based study of 265 hip fracture patients confirmed that MNA-SF predicted mortality, postoperative length of stay, and readmissions, unlike NRS-2002 [71].

The GNRI, calculated using serum albumin and ideal body weight, is efficient and objective. It has proven effective in geriatric orthopedic settings. Its calculation integrates easily measurable parameters, providing a quick and objective assessment of nutritional status, which is especially valuable in cases in which interview-based assessment is not feasible (e.g., for patients with cognitive impairment). It strongly and repeatedly predicts 6- to 24-month mortality and complications in hip fracture cohorts [67,68,70,74,77,88,92,93]. A retrospective study of hip fracture patients found GNRI and CONUT both independently predicted 180-day mortality, with AUCs of 0.74 and 0.72, respectively [88].

The PNI, incorporating serum albumin and lymphocyte count, serves as both an immune and nutritional index. A prospective cohort found a lower PNI score was associated with poorer functional outcomes after hip fracture [94], while a large cohort study reported that patients in the highest PNI category had 39 % lower postoperative complication rates and two-year mortality after hip fracture surgery [95]. Overall, this score shows moderate-to-strong prognostic value for mortality and complications [18,70,94,96,97].

Finally, a recent meta-analysis evaluated MNA-SF, GNRI, PNI, and CONUT across 13 studies, concluding that low GNRI (OR 3.12) and low MNA-SF (OR 3.61) significantly predicted mortality after hip fracture. Due to limited data available on PNI and CONUT scores, it is difficult to draw any strong conclusions for these specific tools [70]. While MNA-SF is effective, simple, and easy to use, its subjective component is a major limitation, whilst PNI, GNRI, and CONUT use objective nutritional indices.

### 3.6. Interventions

Nutritional interventions play a crucial role in the management of older patients suffering from hip fractures. A Cochrane review [98] of 41 randomized and quasi-randomized controlled trials of nutritional interventions for people over 65 years old with hip fractures that included 3881 patients has suggested that oral micronutrients started just before or immediately after surgery can reduce unfavorable outcomes (RR, 0.67) and complication rates (RR 0.69), such as wound infections, pressure sores, and deep venous thrombose. However, it failed to note any changes in mortality, which could be due to a large number of factors affecting mortality after hip fracture like gender, age, fracture type, comorbidity index, cognitive impairment, cardiac anomalies, and pre-fracture mobility.

A more recent systematic review and meta-analysis of 18 randomized controlled trials [99,100,101,102,103,104,105,106,107,108,109,110,111,112,113] involving 1522 older hip fracture patients found similar effects of oral nutritional supplementation (ONS) on the outcomes of these patients [114]. This review showed that ONS significantly improved serum albumin levels and reduced rates of infections, pressure ulcers, and overall postoperative complications. Hospital stay during rehabilitation was also reduced. However, ONS did not significantly affect mortality or readmission rates. Compliance with ONS was generally high (65–100%), but data on adherence factors were limited. This study is the first quantitative meta-analysis to confirm that ONS improves nutritional status and reduces common complications such as infections and pressure ulcers after hip fracture. Given these findings and acceptable compliance rates, the authors advocate for the routine implementation of ONS in perioperative hip fracture care.

The literature increasingly supports that embedding nutritional care within multidisciplinary models enhances the consistency, personalization, and timeliness of interventions, resulting in fewer complications and better recovery trajectories [12,99,103,104,106,112]. Multidisciplinary models integrating dietitian-led nutritional counseling with physiotherapy demonstrate superior intake and lower pressure injury rates [40]. The INTERACTIVE RCT combined individualized exercise and nutrition but yielded no significant gains in gait speed or functional metrics, though protein and energy intake improved [115]. A pilot RCT in femoral fracture patients with sarcopenia combined whey-protein supplementation (0.6–1.0 g/kg/day) and strength exercise, resulting in enhanced grip strength, gait speed, and shorter hospital stays, highlighting the synergistic impact of physical activity and nutrition [116].

The timing of nutritional intervention in older patients with hip fractures is a critical determinant of its effectiveness. Evidence increasingly shows that early initiation—preferably within the first 48 to 72 h postoperatively—yields the most significant clinical benefits. Several randomized controlled trials demonstrated that patients receiving oral nutritional supplements within 48 h of surgery had significantly lower mortality rates and better functional recovery rates than controls [99,103,106,112,117]. The benefits included earlier mobilization and a better functional outcome, a reduced LOS, and fewer infections.

A recent study published in 2023 emphasizes the weak translation of existing evidence into clinical care, citing heterogeneous study designs, the lack of mechanistic evidence to serve as a basis explaining how supplementation affects physiologic and metabolic processes, the cost-effectiveness of nutrition programs, the lack of high-quality RCTs, and inconsistencies regarding supplement composition, dose, and duration [118]. Iatrogenic sarcopenia due to hospital bed rest and insufficient intake during postoperative recovery remains a pervasive challenge, needing integrated solutions.

## 4. Discussion

This systematic review demonstrates that malnutrition is highly prevalent in older patients with hip fractures. Most studies report rates between 15% and 40%, with extremes from 8% up to 64% depending on the assessment tool and timing of evaluation. Pathophysiologically, protein-energy deficits and accompanying sarcopenia compound bone demineralization, impair muscle repair, and foster a hypercatabolic state that impedes healing. Cognitive impairment further amplifies these risks by delaying the recognition and management of nutritional deficits. Importantly, poor nutritional status correlates with fracture type: low lean mass and hypoalbuminemia predispose patients to intracapsular femoral neck fractures, whereas a higher BMI and soft-tissue thickness modulate impact forces in trochanteric fractures. Clinically, malnutrition independently predicts worse postoperative outcomes: it doubles or triples the risk of complications (e.g., infections and delirium), extends hospital stays by 3–7 days on average, and elevates both short- and long-term mortality (a two- to three-fold increase at one year). Functional recovery is similarly compromised, with malnourished patients achieving lower scores on the Barthel Index, slower gait return, and higher rates of institutionalization. Interventional trials, particularly those administering oral nutritional supplementation within 48–72 h postoperatively, demonstrate significant reductions in complications, improvements in serum albumin and biochemical markers, and shorter rehabilitation stays, though effects on long-term mortality and functional endpoints remain mixed. Collectively, these findings underscore the critical need for routine early nutritional screening and timely, targeted supplementation in orthogeriatric care pathways.

Previous reviews and guidelines have long recognized the association between malnutrition and adverse outcomes in hip fracture patients. While earlier meta-analyses and cohort studies focused primarily on single outcomes such as mortality or length of stay and often on one or two assessment tools, the present review synthesizes a broader evidence base spanning prevalence, pathophysiology, fracture mechanics, clinical outcomes, diagnostic instruments, and interventional strategies. Unlike narrower publications, it integrates biomechanical models explaining fracture-type vulnerability, delineates the multifactorial mechanisms (inflammation, anabolic hormone decline, oxidative stress, and cognitive factors), and evaluates both subjective (MNA and SGA) and objective (GNRI, PNI and CONUT) screening methods, highlighting their relative prognostic accuracy. By encompassing 92 studies, this review offers a uniquely comprehensive perspective that aligns with and extends ESPEN’s geriatric nutrition guidelines [14]. Additionally, the explicit focus on the timing of nutritional assessments (within 48 h versus later) addresses a notable gap in the earlier literature, providing actionable insights for clinical practice. Finally, by contextualizing nutritional strategies within multidisciplinary models combining dietetic counseling, resistance exercise, and pharmacologic adjuncts, this review identifies synergistic approaches that were underrepresented in prior analyses, thereby strengthening the evidence for integrated orthogeriatric care pathways.

This review’s narrative design, while enabling a broad thematic synthesis, precluded quantitative meta-analysis due to heterogeneity in study designs, populations, outcome measures, and intervention protocols. The variability in diagnostic criteria, ranging from different MNA cut-offs to diverse biochemical markers, limits direct comparability across studies. However, by grouping findings according to the screening tool used and clearly reporting the prevalence ranges and prognostic validity per instrument, the review minimizes the impact of these inconsistencies. Another limitation is the mixed quality and size of interventional trials, of which some were retrospective, lacked blinding, or had small sample sizes, which may account for inconsistent long-term mortality and functional outcomes. Yet, the consistency of short-term benefits across multiple randomized and quasi-randomized studies adds robustness to the conclusion that early oral nutritional supplementation reduces complications and improves biochemical status. The predominance of studies from high-income countries may restrict generalizability to lower-resource settings; nonetheless, the underlying mechanisms of malnutrition and catabolism are universal, and the review highlights the need for context-specific RCTs globally. Finally, the reliance on published English-language studies introduces publication bias, but this paper clearly identifies evidence gaps such as optimal supplement composition, cost-effectiveness assessments, and mechanistic RCTs to inform future research priorities.

## 5. Conclusions and Future Directions

Malnutrition is a modifiable but often under-recognized determinant of poor prognosis in older patients with hip fractures. Its presence is strongly associated with worse surgical outcomes, increased complications, prolonged rehabilitation, and higher mortality. Despite growing evidence supporting the benefits of nutritional intervention, routine nutritional screening and treatment are not yet universally adopted in clinical practice. The integration of personalized, multidisciplinary nutritional strategies into standard hip fracture protocols is essential to improve functional outcomes, reduce complications, and enhance the quality of life of this vulnerable population.

Future research should focus on the following: (i) standardizing **diagnostic criteria** for malnutrition using validated tools like MNA-SF, GNRI, or PNI to allow for consistent comparison and intervention timing; (ii) conducting high-quality **randomized controlled trials** to assess the optimal composition, dosage, and duration of supplementation in hip fracture patients; (iii) investigating **cost-effectiveness** and implementation strategies of routine nutritional support in orthogeriatric care pathways; and (iv) exploring the **interaction between nutrition, sarcopenia, and physical rehabilitation**, including combined nutrition–exercise programs tailored to patient frailty levels.

## Figures and Tables

**Table 1:** Comparison of malnutrition screening tools in elderly hip fracture patients.

Tool	Components	Scoring and Interpretation	Strengths	Limitations
**MNA**	- BMI - Recent weight loss - Appetite - Mobility - Stress/disease - Psychological issues - Calf circumference (Full version includes more detail)	MNA-SF: 12–14 = Normal 8–11 = At risk 0–7 = Malnourished	- Validated in older adults - Quick (short form) - No lab tests needed	- Some subjective elements - Less precise in acute illness
**GNRI**	- Serum albumin - Current weight vs. ideal weight - GNRI = (1.489 × albumin [g/L]) + (41.7 × (weight/ideal weight))	>98 = No risk 92–98 = Low 82–91 = Moderate <82 = Major risk	- Simple - Objective - Ideal for elderly - Uses routine lab data	- Not usable without lab values - Requires height/weight history
**NRS-2002**	- BMI - Weight loss - Reduced intake - Disease severity - Age > 70	Score ≥ 3 = Nutritional risk	- Validated in hospital settings - Recommended by ESPEN	- Requires clinical judgment - Some complexity in scoring
**PNI**	- Serum albumin - Lymphocyte count - PNI = (10 × albumin [g/dL]) + (0.005 × lymphocytes [/mm^3^])	>45 = Normal 40–45 = Mild risk <40 = Severe risk	- Prognostic in surgical/oncology patients - Simple formula	- Not tailored for geriatrics - Limited functional info
**CONUT**	- Serum albumin - Total cholesterol - Lymphocyte count	0–1 = Normal 2–4 = Mild 5–8 = Moderate 9–12 = Severe	- Fully objective - Easy to automate - Prognostic value	- Requires lab results - Not validated in all populations

MNA = Mini Nutritional Assessment; GNRI = Geriatric Nutritional Risk Index; NRS-2002 = Nutritinal Risk Screening 2002; PNI = Prognostic Nutritional Index; CONUT = Controlling Nutritional Status.

## Data Availability

Not applicable.
